# Report of the First Meeting of the Middle East and Eastern Europe Rabies Expert Bureau, Istanbul, Turkey (June 8-9, 2010)

**DOI:** 10.4061/2011/812515

**Published:** 2011-09-12

**Authors:** Orhan Aylan, Aly Fahmy Mohamed El-Sayed, Firouzeh Farahtaj, Ali R. Janani, Olga Lugach, Olgha Tarkhan-Mouravi, Gaye Usluer, Rodovan Vodopija, Nenad Vranjes, Noël Tordo, Betty Dodet

**Affiliations:** ^1^Rabies Diagnosis and Rabies Vaccine Production Laboratory, Etlik Central Veterinary Control and Research Institute, Ahmet Şefik Kolaylı Cad. No. 23, Etlik, Keçiören, 06020 Ankara, Turkey; ^2^Applied Research Sector, Egyptian Company for Biological Products, Drugs and Vaccines (Egy-Vac/VACSERA), 51 Wezaret El Zeraa Street, Agouza, Cairo 22311, Egypt; ^3^WHO-Collaborating Centre for Reference and Research on Rabies, Pasteur Institute of Iran, 69 Pasteur Avenue, Tehran 1316943551, Iran; ^4^Epidemiology Department of the Sanitary and Epidemiology Surveillance Agency, Ministry Health of Ukraine, 01000 Kiev, Ukraine; ^5^Vaccine Preventable Diseases Surveillance Division, National Center for Disease Control and Public Health (NCDC), 9 M. Asatiani street, 0177 Tbilisi, Georgia; ^6^Infectious Diseases and Clinical Microbiology Department, Medical Faculty, Eşkisehir Osmangazi University, Eşkisehir, Turkey; ^7^Dr. Andrija Štampar Institute of Public Health, Zagreb, Croatia; ^8^Epidemiology Department, Pasteur Institute-Novi Sad, Hajduk Veljkova 1, 21000 Novi Sad, Serbia; ^9^Institut Pasteur, Paris, France; ^10^Dodet Bioscience, Lyon, France

## Abstract

Rabies is a threat in all parts of the world where animal reservoirs persists, including Eastern Europe and the Middle East. Rabies experts from seven Middle East and Eastern European countries (Croatia, Egypt, Georgia, Iran, Serbia, Turkey, and Ukraine) met for two days in Istanbul, Turkey (June 8-9, 2010), to exchange information on the epidemiological situation concerning human and animal rabies in their respective countries and to discuss strategies for rabies elimination and control. They decided to establish a regional network, the Middle East and Eastern Europe Rabies Expert Bureau (MEEREB), a regional network of experts, to increase collaboration in rabies prevention and control at the local, regional, and global levels.

## 1. Introduction

Rabies is a major public health problem causing approximately 55,000 human deaths every year, mainly in Asia and Africa [1]. It is also a threat in other parts of the world where animal reservoirs persist, as is the case in Middle East countries. Rabies continues to be a significant but underestimated public health concern in the region and the situation in some countries has worsened, given the deteriorating environment [2, 3]. While rabies rarely occurs in humans in most of Eastern Europe, thanks to post-exposure prophylaxis (PEP), it remains present in animal reservoirs, particularly in countries situated at the crossroad of Asia, Africa, and Western Europe [4, 5]. 

A group of experts in infectious diseases, rabies, and vaccinology from various institutions in seven Middle East and Eastern European countries met for two days in Istanbul. They were invited through the Ministries of Health/national CDCs, or directly through personal contacts. The number of participants/countries was voluntarily limited as the sole aim of this initial meeting was to evaluate the feasibility/interest of establishing a regional network. During this meeting, participants exchanged information on the epidemiological situation of rabies in their respective countries and discussed specific issues and strategies for rabies elimination and control in their region. They focused on human rabies, but the animal rabies situation was also discussed, since prevention of human rabies requires the control of rabies reservoirs in animals.

Participants agreed to establish an informal regional network—the Middle East and Eastern Europe Rabies Expert Bureau (MEEREB)—to complement the Asian Rabies Expert Bureau (AREB), established in 2004 [6–8] and AfroREB, a network of experts from French-speaking Africa, established in 2008 [9, 10].

## 2. The Rabies Situation

The rabies situation presented during the meeting is summarized in Table 1. All countries represented at MEEREB reported animal rabies, and in some countries where canine rabies is prevalent, the disease still occurs in humans. Cats also play a role as a vector especially in Ukraine, where, according to the data presented, 12 out of 29 (41.4%) human rabies cases in the last 10 years were transmitted by cats, and in Turkey where the recent reappearance of the disease in this species is a concern. 

In Croatia and Serbia, no human rabies case has been reported for over 30 years. The last case in Croatia was reported in 1964, and the last case in Serbia was in 1980: rabid dogs caused both. Since then, there have been two-imported cases of human rabies in Croatia. The absence of cases in Serbia and Croatia can be explained by the fact that rabies is present in wildlife only, vaccination of pet dogs is mandatory, and PEP of animal bite victims is accessible free of charge. However, rabies is still enzootic in the red fox in these two countries, and sporadic cases spill over to other wild animal species and domestic animals. In each country, >1,500 animal-bite victims receive post-exposure prophylaxis (PEP) annually. Human rabies immunoglobulins (HRIG) are locally produced by the Zagreb Institute of Immunology and by the Belgrade Blood Transfusion Institute of Serbia. 

The specific rabies situation in *Turkey *was described; dog-mediated urban rabies predominates, with foci in the Istanbul region. However, fox rabies has been increasing since 1999, especially in the western Aegean region where the numbers of rabid dogs and foxes are approximately equal, a situation unique to Turkey. Occasional rabies cases are observed in the jackal, particularly around Istanbul. These data have also been published recently [11]. One to two human cases are reported annually.

*In Iran,* rabies is the most important zoonotic disease and has spread across the country including the central desert areas; the most affected provinces are located in the north-east, east, and south. The country is spending an increasing portion of its health budget on procurement of cell culture rabies vaccines and immunoglobulins to meet the increasing demand for rabies PEP. The number of people receiving PEP in the 300 bite management centres across the country has more than doubled between 1997 and 2009, while the rabies mortality rate has decreased from 0.9 per million people in the 1980s [12] to 0.02-0.03 per million people in recent years.

In *Egypt*, animal rabies is present both in urban areas and rural settlements. Stray dogs are the main transmitters to other animals and humans. The situation has been stable for the last 10 years with an annual number of 80 human rabies cases. Among the countries reporting data at the meeting, it had the second highest human rabies incidence, following *Georgia* where the number of reported admissions for PEP following exposure to potentially rabid animals has been increasing steadily (from ~10,000 in 2000 up to 28,055 PEP in 2008—with a peak of ~48,000 in 2006). 

Several of the countries represented receive support from the European Union (EU) for oral vaccination of foxes (Croatia, Serbia, and Turkey) and dogs (Turkey) [11]. Western Europe eliminated animal rabies through compulsory vaccination of dogs and oral vaccination of wildlife [13], but the persistence of rabies in animals along borders is a permanent threat. This is illustrated by the reemergence in 2008 of animal rabies in Italy in an area bordering Slovenia, and it spread through the north-western provinces [14, 15].

## 3. Rabies Prophylaxis

According to WHO, immediate post-exposure vaccination is recommended for category II exposure (nibbling of uncovered skin, minor scratches, or abrasions without bleeding;) and immediate vaccination and administration of rabies immunoglobulin are recommended for category III (single or multiple transdermal bites or scratches, contamination of mucous membrane with saliva from licks, licks on broken skin, exposures to bats). For categories II and III, thorough washing and flushing (for 15-minutes, if possible) with soap or detergent and copious amounts of water of all bite wounds and scratches should be done immediately, or as early as possible following the bite [16]. Intramuscular vaccination consists of either a 5-dose (1 dose on each of days 0, 3, 7, 14, and 28) or a 4-dose schedule (2 doses on day 0—1 in each of the 2 deltoid or thigh sites—followed by 1 dose on each of days 7 and 21). An intradermal regimen (injection of 0.1 ml at 2 sites—deltoid and thigh—on days 0, 3, 7, and 28) may be used for people with category II and III exposures in countries where the intradermal route has been endorsed by national health authorities [16].

PEP is free of charge for the patient in all countries participating in MEEREB, except Egypt, where rabies immunoglobulin (RIG) is rarely administered, even to patients with category III exposure. In Georgia, PEP is free of charge for children under 18 years of age and adults without social coverage. Modern cell culture rabies vaccines are used intramuscularly (IM) for pre- and post-exposure prophylaxis. Most countries use 5-dose IM regimen (at days 0, 3, 7, 14, and 28). In three countries (Egypt, Georgia, and Ukraine), a 6-dose IM vaccination schedule (with an additional dose at day 90) is applied in compliance with the labeling of some regionally produced rabies vaccines. 

Human rabies immunoglobulin (HRIG) is used in the public sector in Serbia, Croatia, and Iran. In the other countries, it is available in the private sector only, while equine rabies immunoglobulin (ERIG) is used in the public sector and produced in Ukraine for local use.

Pre-exposure prophylaxis (PrEP) is administered free of charge to people at high risk of exposure in most MEEREB countries, as recommended by WHO [16]. The exceptions are Georgia and Egypt, where it is provided on a voluntary basis. Those vaccinated pay for the treatment.

## 4. Discussion

This meeting was a first step in establishing a rabies network in the Middle East and Eastern Europe. During this initial meeting, participants noted the common issues and differences in their respective rabies situation and discussed how they could benefit from sharing their experiences to establish a regular, supranational collaboration to fight rabies.

It was noted that data on human rabies cases are available at the national level, but that they need to be consolidated and made available at the international level. For instance, 2 and 6 human cases were reported to have occurred in 2009 in Turkey and Ukraine, respectively (Table 1), while none was reported to the Rabies Bulletin Europe [17].

Human cases still occur in countries where rabies is present in dogs, in spite of vaccine and RIG availability. Strengthening dog vaccination and dog population control is key to rabies control. It was reported at the meeting that among the 1-1.2 million privately-owned dogs in Iran, approximately 400,000 are vaccinated, while there is no vaccination programme for unowned and stray dogs, the estimated number of which is about 1 million [12]. This results in an estimated 15–20% vaccination coverage for the dog population, which is insufficient, since vaccinating at least 70% of the whole dog population is necessary for dog rabies control [18]. The situation is even less clear for cats. 

Therefore, the Blueprint for Rabies Prevention and Control that was previewed during the MEEREB workshop was enthusiastically welcomed by the participants as it helps and guides those implementing rabies control programmes for dog-rabies elimination with a detailed step-by-step action plan. This paper has been prepared by a global group of rabies experts (Partners for Rabies Prevention) under the leadership of the Global Alliance for Rabies Control (GARC) and is available online and can be downloaded free of charge [19].

Furthermore, MEEREB participants noted the need to increase rabies awareness in their region, and agreed to participate in the World Rabies Day. They also noted the need for timely information for people travelling to rabies enzootic areas (i.e., travellers to their regions and/or people from their regions to other enzootic parts of the world). Recent studies showed that most animal-associated injuries requiring PEP in French travellers occur during visits to Thailand and Turkey—countries for which travellers do not usually seek advice since they are not associated with more conventional travel-associated diseases like malaria or yellow fever [20]. The majority of travellers bitten by animals do not receive adequate PEP, or else they experience a substantial delay before receiving it [20–23]. 

The participants agreed to meet regularly and to invite experts from other countries of the region to join the MEEREB network, and participate in the next MEEREB workshop.

## Figures and Tables

**Table 1:** Rabies epidemiology and management in the 7 countries represented at MEEREB.

	Croatia	Egypt	Georgia	Iran	Serbia	Turkey	Ukraine
Main reservoir(s)	Fox	Dog	Dog, jackal, wolf	Dog, wolf, fox, jackal	Fox	Dog, fox, jackal	Dog, fox

Main vector(s)	Dog, cat	Dog	Dog, cat	Dog	Dog, cat	Dog	Cat, dog

Human population (estimates)*	~4,500,000	~80,500,000	~4,600,000	~76,900,000	~7,300,000	~77,800,000	~45,500,000

Number of human rabies cases reported between 2000–2009 [1999–2009 for Turkey]	0	880	90	62	0	21	29

Human rabies cases reported in 2009**	0	80	6	2	0	2	6

Estimated human rabies incidence per million (2009)**	—	0.99	1.30	0.02	—	0.025	0.13

Number of PEP (2009)	1,750	~200,000	28,055	130,531	1,609	178,250	21,000

PEP incidence per million people (2009)**	388	~2,485	6,100	1,700	220	2,290	461

PEP vaccination regimen	4 doses IM (Zagreb 2-1-1) Rarely: 5 doses IM (Essen)	6 doses IM *at days 0, 3, 7, 14, 28, 90 *	6 doses IM *at days 0, 3, 7, 14, 28, 90 *	5 doses IM *at days 0, 3, 7, 14, 28 *	5 doses IM *at days 0, 3, 7, 14, 28 *	5 doses IM *at days 0, 3, 7, 14, and 21 or 28 *	6 doses IM *at days 0, 3, 7, 14, 28, 90 *

Type of rabies Immunoglobulin used	HRIG (locally produced)	HRIG (private sector)	ERIG (public sector) HRIG (private sector)	HRIG (public sector)	HRIG (locally produced)	ERIG (public sector) HRIG (private sector)	ERIG (locally produced)

Number of PrEP in 2009	200 (population at risk)	N.a.***	Vets (on a voluntary basis)	N.a. (population at risk)	51 (population at risk)	991 (population at risk)	N.a. (population at risk)

Figures reported by MEEREB participants.

*July 2010 estimates—The World Fact Book—https://www.cia.gov/library/publications/the-world-factbook/.

**Calculated from data reported by participants for the population mentioned.

***N.a.: data not available.
